# Using genomic screening methods to create high-quality rice breeding material

**DOI:** 10.18699/vjgb-26-63

**Published:** 2026-07

**Authors:** N.G. Tumanyan, Zh.M. Mukhina

**Affiliations:** Federal Rice Research Centre of the Ministry of Agriculture of the Russian Federation, Belozerny, Krasnodar, Russia; Federal Rice Research Centre of the Ministry of Agriculture of the Russian Federation, Belozerny, Krasnodar, Russia

**Keywords:** rice, selection, genotyping, sequencing, marker, rice quality, vitreousness, grain shape, рис, селекция, генотипирование, секвенирование, маркер, качество риса, стекловидность зерна, форма зерна

## Abstract

Rice, as a key model in the study of agroecosystem genomics, is the focus of research meant to address the challenges of producing sufficient food for the growing global population. In breeding programs developing new varieties, improving the physicochemical properties of the grain is crucial. Based on the analysis of national and international research, this article presents information on new molecular genetic methodologies and advances in the development of new valuable rice genotypes using genome sequencing data. Continuous enrichment of rice germplasm at global breeding centers is achieved through the use of highly effective approaches employing postgenomic and cellular technologies in combination with traditional phenotyping methods. This review examines the achievements of molecular genetic research in rice, focusing on valuable grain quality traits such as vitreousness (chalkiness) and shape (size). GWAS analysis is widely used in marker-assisted and genomic rice breeding programs. More recently, GBS analysis has been used to identify relationships between phenotype and genotype based on the analysis of bi-parental mapping populations and varietal accessions. The post-genomic research period, focused on the search for candidate genes for valuable quality traits, had started after the genomic reference sequences were obtained. As a result, hundreds of QTLs for the chalkiness trait were discovered across 12 chromosomes, few were accurately mapped or sequenced. By 2018, several major QTLs affecting grain size were sequenced and characterized. For example, the presence of the recessive GS3 allele and the dominant GW7TFA allele increases the grain length-to-width ratio. In 2023, it was shown that overexpression of OsFIF3 inhibits the expression of FLO2 and SUT1, thereby increasing chalkiness and reducing grain size. This breeding breakthrough is attributed, for example, to the use of non-digital markers for length, width, thickness, and the grain length-to-width ratio, GS3RGS1 and RM505, as selection markers. All research is an ongoing process aimed at achieving the highest possible level of high-quality rice products.

## Abstract (ru)

Рис как ключевая модель в изучении геномики агроэкосистем находится в центре внимания исследований по решению задач производства достаточного количества продуктов для растущего населения Земли. Качество зерна возделываемых сортов риса лежит в основе его конкурентоспособности и культуры потребления. На основе анализа отечественных и зарубежных исследований представлены сведения о молекулярно-генетических методах и достижениях в создании новых ценных генотипов риса при использовании данных секвенирования геномов. Непрерывное обогащение генплазмы риса в мировых селекционных центрах происходит благодаря высокоэффективным подходам с применением постгеномных и клеточных технологий в сочетании с традиционными методами фенотипирования. В обзоре рассматриваются достижения молекулярно-генетических исследований риса, относящихся к ценным признакам качества зерна – его стекловидности (мучнистости) и форме (размерам). В программах по маркер-ориентированной и геномной селекции риса широко используется анализ GWAS. В последние годы генотипирование методом GBS стало широко применяться для выявления взаимосвязи между фенотипом и генотипом на основе анализа двуродительских картирующих популяций и выборок сортообразцов. Постгеномный период исследований, связанный с поиском генов- кандидатов ценных признаков качества, начался после получения референсных последовательностей геномов. В результате были обнаружены сотни QTL признака мучнистости по 12 хромосомам, немногие из них были картированы или секвенированы. К 2018 г. секвенировано и охарактеризовано несколько основных QTL, влияющих на размер зерна. Наличие рецессивного аллеля GS3 и доминантного аллеля GW7TFA увеличивает соотношение длины зерна к ширине. В 2023 г. было показано, что сверхэкспрессия OsFIF3 ингибирует экспрессию FLO2 и SUT1, тем самым увеличивает мучнистость зерна и снижает его размеры. Селекционный прорыв связывают с использованием неcпецифических маркеров признаков длины, ширины, толщины, отношения длины зерновки к ширине, GS3RGS1 и RM505 в качестве маркеров выбора. Все исследования представляют собой непрерывный процесс с целью выхода на максимально возможный уровень реализации высоких параметров качества продукции из риса

## Introduction

Rice, a valuable cereal crop, has ranked first or second among cereals in Russia in recent years and is used in both traditional and dietary nutrition. Rice is a staple crop for half the world population, and it is a key model for studying the genomics of agroecosystems, making it a focus of research aimed at solving the problem of producing sufficient food for the growing world population (Wing et al., 2018). To date, the origin of rice has not been definitively established: centers of its cultivation have been discovered in Korea, India, and China.

Global rice consumption is approximately 500 million tons per year, ranging from 0.01–0.9 kg/person in Lebanon and Latvia to 230–280 kg/person in Vietnam and Myanmar, respectively. Approximately 90 % of rice is produced in traditional rice-consuming countries – East and Southeast Asia.

Rice consumption in Russia is approximately 670,000 tons per year. Russian rice production, being a significant contributor to national agricultural production (eight regions produce rice), ensures the country’s food security in terms of rice.

The economic value of rice is determined by the profitability of producing the raw material (rice grain) and rice products. Among the factors determining this, the grain physicochemical properties, including those that contribute to its nutritional and culinary qualities (ECQ), are crucial. Therefore, the grain quality of cultivated rice varieties underlies consumer culture and its competitiveness.

Rice quality parameters required for production and consumption form the basis of breeding programs for developing new varieties. Rice quality is a complex, integrated indicator determined by numerous physical properties of the grain (Alyoshin E.P., Alyoshin N.E., 1997). The absolute requirements that promising rice varieties must meet are high quality performance and compliance with production and consumer requirements. The concept of rice quality includes a large number of morphological, physicochemical and biochemical characteristics, which, as a rule, are the subject of fundamental, applied and interdisciplinary research (Han et al., 2004; Koutroubas et al., 2004; Addison et al., 2015; Odoom et al., 2021).

The set of physicochemical grain quality traits includes a wide variety of parameters assessed in scientific breeding programs and production processes: moisture content, grain calorific value, grain vitreousness, density, hardness, strength, grain cracking, total grain yield, whole kernel content, chaff, bulk density, etc. Morphological traits include size (length, width, thickness), shape (caryopsis length-to-width ratio), weight, awns, pubescence, caryopsis ribbing, and other attributes.

Achievements in international agricultural plant breeding are largely determined by the widespread use of modern biotechnologies in breeding programs, such as whole-genome sequencing, sequencing of plant genome regions within large-scale genotyping, targeted genotyping, haploid technologies, and others.

## Rice grain quality. Vitreousness, grain size and kernel shape

As living standards rise, improving rice quality becomes increasingly important. It is widely recognized that worldwide, improvements in quality lag behind yields. Rice quality is determined by numerous physical properties of the grain. The grain appearance and processing parameters are a priori determined by its basic physical properties (vitreousness/chalkiness, grain shape/size, etc.), which are the primary determinants of rice’s market value.

Rice vitreousness is defined as the percentage of vitreous grains in the grain mass (see the Figure). Rice chalkiness is a highly undesirable grain quality that develops during grain filling and is one of the key indicators of grain appearance. It not only affects the grain appearance but also its physical and chemical properties, polishing quality, and cooking quality (Peng et al., 2014). Due to the disordered structure of starch granules and air spaces, chalky grains easily break during hulling and polishing, reducing the yield of high-quality rice products. Rice grain chalkiness, including white heart, belly and back, refers to the opaque part of rice endosperm caused by loose starch granules, which may depend on both the cultivar potential and its response to natural and anthropogenic factors (Guo et al., 2011; Peng et al., 2014; Du et al., 2023; Gong et al., 2023).

**Fig. 1.**
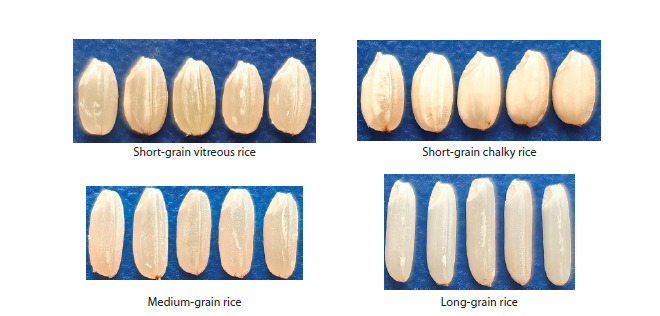
Types of rice by grain vitreousness and grain shape.

Grain size assessment involves determining the length, width, and thickness of the grain in mm; and grain shape, as the ratio of grain length-to-width. Grain size, considered a morphological trait, is a key agronomic characteristic associated with yield and quality. Smallgrain rice can have a grain length of up to 5 mm, while long-grain rice can have a grain length of over 6.5 mm. In Russia, rice breeding categorizes rice varieties as short-grain (with a grain length-to-width ratio, l/b, of up to 2.0), medium-grain (l/b up to 3.0), and long-grain (l/b > 3.0) ones.

Highly vitreous rice is valued more than rice with predominantly chalky grains. However, there are varieties used in traditional Italian and Spanish dishes, such as risotto and paella, that are characterized by a virtually complete absence of vitreous grains.

Therefore, this review aims to present global achievements in genomic rice breeding in terms of rice quality, which have made a significant contribution to the creation of new, sought-after genotypes of cultivated rice (see the Figure).

## Biotechnological methods for enriching the germplasm of agricultural crops

Continuous enrichment of rice germplasm in the world breeding centers is based on the use of highly effective approaches using post-genomic and cellular technologies in combination with traditional phenotyping methods, which ensures the acceleration of the breeding process, high information content of characteristics (traits of interest), and leads to the maximum positive result in the modern realities of the scientific breeding process (Addison et al., 2015; Garkusha et al., 2022).

The genomic approach is very popular abroad in practical agricultural plant breeding projects, and its high cost does not discourage its use due to its high efficiency. The French company Limagrain uses it to develop modern sunflower hybrids, whereas Euralis Semences uses it to develop breeding forms of sorghum crops (grain, sweet sorghum, sorghum-sudangrass hybrids), and Syngenta uses it to develop sunflower, corn, vegetables and other crops. The routine use of genomic screening methods and the creation of breeding material largely determines the breeding success of these companies.

The future of crop breeding is currently linked to the latest advances in crop genome sequencing and assembly technologies (Dmitriev et al., 2022). The development of molecular biology methods for restriction analysis, PCR DNA amplification, and subsequent nucleotide sequence determination by DNA sequencing has already led, at the initial stages, to the use of DNA regions (nucleotide sequences) linked to genes of interest (which determine certain quality traits of an agricultural crop) or located within their structure as a new group of molecular genetic markers.

The use of genome sequencing technologies has led to fundamental results: the identification of genes of interest with the assessment of polymorphism within the species and the development of molecular markers of economically valuable traits (including grain quality traits), on the basis of which marker-assisted selection (MAS) and genomic breeding programs for crops are implemented.

Genome-wide association studies (GWAS) analysis is widely used in marker-assisted and genomic selection programs. The more recent development of GBS (genotyping-by-sequencing) has been used to identify relationships between phenotype and genotype based on the analysis of bi-parental mapping populations and cultivar samples. This method is based on developed SNP chips (SNPs: single-nucleotide polymorphisms) and genotyping methods using NGS sequencing (NGS: next-generation sequencing, a short-read sequencing technology) of selected genome fractions. The resolution of GWAS can reach up to a single nucleotide. Candi- date genes are identified, validated, and tagged (Baxla et al., 2025). The large-scale use of high-throughput sequencing (GBS) genotyping technology has been successfully applied to agricultural plants, as confirmed by numerous publications in the international scientific literature.

Genomic selection (GS) used in agricultural crop breeding offers advantages over marker-assisted selection for polygenic selection. While MAS is effective for monogenic trait control, GS involves analyzing a training population using phenotyping and genotyping data, identifying correlations between phenotype and genotype, and selecting genotypes from a set of selection candidates. The optimal number of training populations, markers, and their ratios are key to successful breeding. The introduction of accelerated genomic technologies – GS, NGS, SNP-mediated selection (using markers and GWAS), with accompanying phenotyping and bioinformatics – is already widely used in modern breeding, leading to the creation and use of new crop genotypes in agriculture.

The development of highly efficient sequencing technologies and the acquisition of reference sequences of plant genomes has led to a “post-genomic” period of research: within this period, an effective search for candidate genes of economically valuable traits, new markers, and the use of the obtained data in genomic selection are being carried out.For the first time in Russia, scientists from Skoltech, together with colleagues from the University of Southern California, the Vavilov Federal Research Center, the Pustovoit All-Russian Research Institute of Oil Crops, and the Agroplasma breeding and seed company, conducted large-scale genotyping of a sunflower collection using the Illumina platform. The authors conducted the genetic analysis and phenotyping of 601 cultivated sunflower lines, which enabled bioinformatics analysis to identify genetic markers for monitoring the fatty acid composition of the oil (Goryunova et al., 2019). Within the framework of the grant from the Russian Science Foundation and the Kuban Science Foundation (22-16-20015), phenotyping of samples of segregating rice populations with different amylose content and grain color was carried out in order to select SNP candidate markers for rice breeding based on amylose content, followed by sequencing of DNA libraries on an Illumina MiSeq device (Mukhina et al., 2024).

A development by scientists from Novosibirsk is of great importance for the use of biotechnology in breeding programs. Through the efforts of scientists from the Institute of Cytology and Genetics of the Siberian Branch of the Russian Academy of Sciences, the Kurchatov Genome Center of the Institute of Cytology and Genetics of the SB RAS, the Novosibirsk State Agrarian University, and Novosibirsk State University, the GBS-DP bioinformatics pipeline was developed based on the Snakemake process management system. The program enables fully automated GBS data analysis, including the installation of necessary software packages and the processing of large datasets (over 400 samples) (Pronozin et al., 2023).

At the Federal State Autonomous Educational Institution of Higher Education “Peter the Great St. Petersburg Polytechnic University”, St. Petersburg, an SNP panel was developed from a set of molecular markers based on single-nucleotide polymorphisms of the soybean genome with the help of which genotypes are identified. It makes it possible to assess the genomic value of soybean breeding material and conduct soybean breeding for a set of economically valuable traits: variety/line productivity, oil and protein content in seeds (Samsonova et al., 2021).

International research on genomic approaches in practical crop breeding projects, including rice, is of interest due to the importance of incorporating the latest global advances into the breeding process for developing national varieties.

Rice is a model plant for studying and understanding aspects of crop genomics and phenomics. The first crop for which the first reference genome sequence was published using NGS technologies (2002) was rice (Oryza sativа). Subsequent application of the latest sequencing technologies, which produce reads of 20,000 nucleotide pairs, resolved the problems of polyploidy and high repeat content. As early as 2010, information was published on a whole-genome analysis of 1,536 SNPs using Illumina technologies for genotyping 395 diverse accessions of rice (O. sativa).

The analysis was developed based on a subset of SNPs detected using Perlegen hybridization technology and served to validate the majority of the detected SNPs in a high-quality MBML-intersect dataset (Zhao K. et al., 2010). The technology was used on crops such as barley (Yao et al., 2018), wheat varieties and hybrids (Tyrka et al., 2021), Brassica napus L., Ralstonia solanacearum, Papaver bracteatum (Rahmati et al., 2024), Vigna radiata L. (Kumari et al., 2023), B. napus L. (Kim еt al., 2024), Logan (Thai fruit of the genus Dimocarpus) (Riangwong et al., 2023), Eucommia ulmoides (grown in China) (Liu C. et al., 2022), Cucumis sativus L. (Kishor et al., 2021) and many others in China, South Korea, USA, India, Iran and other countries.

Results from sequencing and resequencing crop genomes have enabled the identification of SNPs and the development of SNP chips used for genotyping multiple genotypes. RNA-seq and exome sequencing technologies also enable the identification of new SNPs.

In the field of genotyping, works on high-throughput sequencing of rice are known by scientific teams from China (Zhu et al., 2018; Eltahawy et al., 2020; Wang Y. et al., 2020; Yuyu et al., 2020; Pan et al., 2021; Yang W. et al., 2021b; Li J. et al., 2022; Du et al., 2023; Gong et al., 2023; Jin et al., 2023; Tang et al., 2023; Wang N. et al., 2023; Wu et al., 2023; Gao J. et al., 2024; Liu J. et al., 2024; Xu et al., 2024; Yang W. et al., 2024), India (Naidu et al., 2017; Ngangkham et al., 2018), the USA (Zhao K. et al., 2010; Kumar et al., 2023), the Philippines (Cuevas et al., 2016), Vietnam (Piao et al., 2023), Indonesia (Susiyanti et al., 2020), Israel (Heifetz, Soller, 2015); international teams from the Philippines, China and Germany (Sreenivasulu et al., 2022), China, the Philippines, France (Qiu et al., 2015), Uruguay, the USA, Argentina (Quero et al., 2018), India and the USA (Kumari et al., 2023), Egypt and China (Ali et al., 2020), etc.

The vast amount of rice genome sequencing and resequencing data has enabled the development of databases for a variety of purposes, including the Genome Variation Map (Song et al., 2018), RiceVarMap (Zhao H. et al., 2015), SNP-Seek database (Alexandrov et al., 2015), RPAN and MBK V1 (Institute of Genetics and Developmental Biology, CAS, 2018).

Based on the sequencing data of 3,010 rice genomes, tools for searching and visualizing rice pan-genomes were developed: Rice Pan-genome Browser (RPAN, http://cgm.sjtu.edu.cn/3kricedb/) and Rice Functional and Genomic Breeding (RFGB, http://www.rmbreeding. cn/Index/), containing genomic sequences, gene annotations, expression data, and PAV (presence/absence variations). More than 12,000 genes absent from the rice reference genome were included (Sun et al., 2017; Wang H. et al., 2020).GBS technology was successfully used for genetic determination of the “days to flowering”, “plant height”, and “panicle productivity” traits under drought stress in two mapping rice populations obtained from crossing the Swarna*2/Dular and IR11N121*2/Aus 196 varieties (Yadav et al., 2019). As a result of genotyping using a custom marker panel of the segregating population of F2 rice of the Nipponbare variety with wild African rice O. longistaminata, 8,154 informative SNP markers were identified and quantitative loci (QTL) responsible for the “panicle number” trait were localized on chromosomes 1, 3, 4, and 8 (Furuta et al., 2017).

Genome editing technology (first used in plants in 2013) based on the CRISPR/Cas9 system (a multiplex genome editing system) allows for point mutations and the insertion and deletion of genes in specific regions of the genome. This breakthrough technology is being used extensively in rice. It has already been applied to multiple target genes in rice, improving the grain quality of breeding material, producing non-transgenic plants, and rapidly introducing genetic diversity into crop breeding.

In 2017, Chinese researchers reported the creation of a vector targeting eight rice agronomic genes using CRISPR/Cas9. Further genetic transformation and DNA sequencing revealed high mutation efficiency of the eight target genes, including in subsequent generations of various gene-editing-associated phenotypes in both homozygous and heterozygous states. These results demonstrate the potential of the CRISPR/Cas9 system for rapidly introducing genetic diversity into crop breeding (Shen et al., 2017).

Inactivation of the corresponding enzymes in this type of transformation made it possible to increase or decrease the amylose content, significantly reduce the content of cadmium ions in the grain; it also led to an increase in the content of stable oleic acid in the grain and imparting aroma (Lu, Zhu, 2017; Sun et al., 2017; Hua et al., 2019; Khlestkina et al., 2019).

## Vitreousness/chalkiness traits in rice molecular breeding

Grain appearance is directly related to its properties: grain shape, size, and vitreousness/chalkiness (Guo et al., 2011). Over the past 20 years, international researchers have made significant progress in identifying QTL genes associated with key traits, including appearance, aroma, texture, and nutritional properties. Markers derived from these genetic discoveries have become effective breeding tools for improving rice quality.

Chalkiness trait of rice grain, caused by loose starch granules in the endosperm, reduces its quality and negatively affects the production of rice products. At numerous rice centers in China, the nature of chalky endosperm formation and grain shape have been a key focus of research over the past 10 years. Hundreds of QTLs for the chalky grain trait have been identified. Due to the genetic complexity and instability of the rice chalky grain trait, only a few have been accurately mapped or sequenced to date. Chalk5, a positive regulatory factor particularly expressed in the endosperm and located on chromosome 5 encoding vacuolar pyrophosphatase translocating H++, is the first major QTL sequenced for chalky grain. It influences the grain transport system by affecting pH, and also affects the subcellular ultrastructure of the endosperm and the formation of chalky spots (Li Y. et al., 2014).

In addition, several QTLs, such as qPGWC-7 (Zhou et al., 2009), qPGWC-8 (Guo et al., 2011), and qACE-9 (Gao F. et al., 2016), have been fine-mapped, which is useful for gene sequencing and functional analysis. The QTL for the percentage of chalky grain (PCG), qPCG1, was localized to a 139 kb region on the long arm of chromosome 1 using segregating populations generated from residual heterozygotes of the Xieqingzao B (XB)/ Zhonghui 9308 (9308) cross. The 9308 allele reduced PCG but had little effect on major agronomic traits, which can be used to improve grain appearance without reducing yield (Zhu et al., 2018).

A large number of QTLs for chalky spots in rice grain have been reported across all 12 chromosomes, but few of them have been fine-mapped or sequenced. In 2021, two QTLs for chalky spots were fine-mapped in two single-segment substitution lines (SSSL): 11-09 with a substituted segment from O. sativa and HP67-11 with a substituted segment from O. glaberrima (Yang W., 2021a).

For single nucleotide polymorphisms, 3,830 markers were mapped to 12 linkage groups with a total length of 2,456.4 cM with an average genetic distance of 0.82 cM. Seven grain quality parameters, including transparency (vitreousness), chalky grains content, and relative chalky spot area per grain, were examined in the F2 population. A total of 15 QTLs with logarithm of odds (LOD) scores >4 were identified and mapped to chromosomes 6, 7, and 9. These loci include four QTLs for vitreousness and four for chalky content. Of these, only one matched previously published QTLs, while the rest were novel. By comparing the major QTL regions in the rice genome, several key candidate genes reported to play a crucial role in determining grain characteristics were identified. These results will accelerate the fine mapping of these QTLs and pyramiding of QTLs, which will contribute to the genetic improvement of rice quality (Jin et al., 2023).

At the Rice Research Institute, Anhui Academy of Agricultural Sciences and the College of Agronomy, Anhui Agricultural University (Hefei, China), a genomewide association analysis of the degree of endosperm chalkiness (DEC) and percentage of grains with chalkiness (PGWC) was performed by combining 1.2 million SNPs with phenotypic data from 173 rice accessions and identifying thirteen QTLs for DEC and nine for PGWC (Xu et al., 2024).

Scientists from the Guangdong Provincial Laboratory of Molecular Plant Breeding, the State Key Laboratory of Subtropical Agricultural and Bioresource Conservation and Utilization of South China Agricultural University, and the Guangdong Laboratory of Modern Agriculture (Guangzhou) used six QTLs with low chalkiness to generate 17 pyramided lines with 2–4 QTLs. The results showed that chalkiness decreased with an increasing number of QTLs in the pyramided lines. It was concluded that pyramiding QTLs of genotypes with low chalkiness can be used to develop high-quality rice varieties with high grain vitreousness (low chalkiness) (Yang W., 2024).

The previously described QTL for the chalkiness trait qWCR4 was confirmed in the segregating BC5F2 population and mapped to a 35.26 kb region (Shi et al., 2022).

## Grain size and shape traits in rice molecular breeding

Grain shape is determined by three traits: grain length, width, and thickness, and simultaneously affects rice yield and quality. Although a large number of major genes regulating grain shape have been sequenced in recent years, many minor genes remain to be discovered.

Grain size is one of the key agronomic traits associated with yield and quality. The major quantitative trait loci GS3 and GL3.1 have been shown to play a predominant role in negatively regulating grain size (Yuyu et al., 2020). By 2018, more than 8,500 QTLs regulating various agronomically important rice traits, including grain size, had been mapped using different contrasting segregating populations derived from different parents, including several major QTLs affecting grain size and yield in rice that were sequenced and characterized (Ngangkham et al., 2018).

Association mapping based on 18,824 high-quality markers yielded 38 QTLs for 10 traits, five of which corresponded to known genes or precisely mapped QTLs (Qiu et al., 2015; Quero et al., 2018).

The results obtained by 2019 laid the foundation for studying grain length biodiversity and molecular breeding of different grain types. Analysis of the secondary F2 population from a cross between the Nipponbare and Z741 rice varieties allowed the identification of 20 QTLs for important agronomic traits. The grain length of Z741 was shown to be controlled by a major QTL (qKL3) and a minor QTL (qKL7). Candidate gene sequencing revealed that qKL3 is a possible allele of OsPPKL1, encoding a protein phosphatase involved in brassinosteroid signaling, and qKL7 is an unspecified QTL; eight QTLs (qKL3, qKL7, qRLW3-1, qRLW7, qPH3-1, qKWT3, qKWT7, and qNPB6) were confirmed using three selected singlesegment substitution (SSSL) lines S1, S2, and S3; five QTLs (qKL6, qKW3, qKW7, qKW6, and qRLW6) were detected in S1, S2, and S3 that were not detected in the Nipponbare/Z741 F2 population. Minor QTLs are likely influenced by environmental factors, since QTLs qNPB3, qNPB7, and qPL3 were not validated by three SSSLs (Wang H. et al., 2020).

A study of the genetic effects of grain shape, amylose content, and other quality traits at the Laboratory of Crop Genetic Improvement and Biotechnology and the Key Laboratory of Rice Genetics and Breeding of the Guangxi Academy of Agricultural Sciences revealed that the gs3, GW7, TFA, gw8, and chalk5 genes improve rice appearance. The presence of the gs3, GW7, and TFA alleles increases the rice grain length-to-width ratio, whereas the addition of the gw8 allele can further reduce grain width.

## Genome-wide association studies simultaneously determining size and vitreousness/chalkiness in rice

Much attention is paid to genome-wide association studies of grain appearance (size, shape, chalkiness, content of chalky grains in the total mass) (Zhao D. et al., 2022) of the world collection of rice germplasm and historical breeding populations.

Eight marker–trait associations were identified by screening 124 genotypes with primers specific for grain size traits. Specific markers for length, width, thickness, and grain length-to-width ratio, GS3RGS1 and RM505, have the potential to be used as selection markers in rice breeding (Naidu et al., 2017).

A low correlation with chalkiness was demonstrated at grain widths <2.0 mm, with minimal differences between the Chalk5 and chalk5 alleles (the gene regulating chalkiness development in rice) (Gao J. et al., 2024). Scientists from the Guangxi Agricultural Academy (China) and others have shown that OsMKK3 encodes a kinase of MAP-kinase that controls grain size and chalkiness by influencing cell proliferation in the spikelet coat; Os- SPL16, GS5, and GIF1 have a significant impact on the OsMKK3-regulated grain size pathway (Pan et al., 2021).

A set of recombinant inbred lines (RILs) derived from Gang46B (G46B) and K1075, a G46B introgression line with lower PGWC, was developed. Based on a linkage map containing 33 simple sequence repeat (SSR) markers, a total of 15 additive QTLs were detected, determining six quality traits, including chalkiness and grain shape (Gao F. et al., 2016). A new QTL, qGL11, was identified, potentially encoding cyclt1, which plays a specific role in regulating grain length (Li J. et al., 2022).

Researchers at Hunan Agricultural University conducted DAP-seq of multiple rice accessions and showed that overexpression of OsFIF3 inhibits the expression of FLO2 and SUT1, thereby increasing grain chalkiness and reducing grain size (Tang et al., 2023).

In 2024, it was reported that using backcrosses between the Cypress and KY131 populations, a genetic linkage map was constructed with 151 polymorphic markers, and QTL mapping analysis identified 37 QTLs for grain shape, chalkiness, and amylose content. Among these, three QTLs for grain shape (qGW3, qGL7.2, and qLWR7.1) and four QTLs for grain chalkiness (qWBR1.2, qBR2, qWCR3, and qWCR11) demonstrated repeatable and detectable genetic effects across populations. Consequently, LOC_Os03g45210 may be a candidate gene for qWCR3.

The genetic effects of three grain shape QTLs, qGL7.2, qLWR7.2, and qGW3, and three chalkiness QTLs, qBR2, qWCR3, and qWCR11, were then confirmed in a random population. Using the progeny analysis method, the qWCR3 gene was precisely mapped to a 100-kb region on chromosome 3. The results of this study provide a basis for map sequencing of these QTLs (Liu J. et al., 2024).

Two closely linked quantitative trait loci (QTLs) controlling chalkiness and grain shape were identified on rice chromosome 8 using single-segment substitution lines (SSSLs) (Yang W. et al., 2021b).

In 2023, the results of a study on high-resolution quantitative trait loci mapping using sequencing to determine rice grain quality characteristics, with a particular focus on chalkiness, were published. A genome-wide association study of rice grain shape and chalkiness was conducted using accessions from the Xi’an collection (China). A panel of 137 markers revealed significant genetic variation in six grain quality traits. Twenty-seven QTLs were identified using GWAS. The authors believe that the obtained results improve the understanding of the genetic basis of the physical traits of grain mass, shape, and chalkiness in rice and can be used to provide recommendations for future breeding using markers with QTLs or gene pyramiding to stabilize and improve rice quality (Wang N. et al., 2023).

A total of 185 recombinant inbred F12 lines (RILS), obtained in the United States by crossing Cypress (HNTtolerant) and LaGrue (HNT-sensitive), were screened for grain quality traits: grain length, grain width, and relative chalky grain content under control and stress conditions. A total of 15 QTLs were identified on nine chromosomes, which were scanned for natural genetic variation in the Japanese Rice Diversity Panel (JDP) to identify candidate genes for these parameters. A total of 6,160 high-impact SNPs were detected (Kumar et al., 2023).

The mitochondria-associated protein PPR (demonstrated in the stable mutant WBG1) regulates grain chalkiness and size (Wu et al., 2023).

The RGA1 gene, which encodes the α-subunit of the rice G protein, plays a key role in regulating rice structure, seed size, and response to abiotic stress. Research by Chinese scientists has shown that the RGA1 gene is involved not only in controlling rice structure and grain size but also in regulating rice quality and seed germination (Yang X. et al., 2024).

Recently, 25 quantitative traits associated with the grain length, width and chalkiness under heat stress, or three sets of overlapping QTLs located on chromosomes 3, 5 and 11, were identified, which can be attributed to the priority candidate genes for QTLs associated with the quality of grain appearance (size and chalkiness under heat stress). Mutants LOC_Os05g06920, LOC_ Os05g06970 and LOC_Os11g28104 had an increased grain length and grain length-to-width ratio, as well as a decreased grain width, including under heat stress. Mutants LOC_Os05g06970 and LOC_Os11g28104 significantly reduced grain vitreousness and transparency (Chen L. et al., 2025).

In summary, previous studies have shown that numerous grain quality-related genes affect both grain size and chalkiness in rice (Yang W. et al., 2021b; Zhao D. et al., 2022; Tang et al., 2023; Yang X. et al., 2024). For example, OsMKK3, OsSPL16, GS5, GIF1 (Pan et al., 2021). Several genes responsible for grain shape, including gw8, GW7, lgy3, and gs9, can reduce starch content and significantly improve the grain appearance of rice (Liu Q. et al., 2018; Zhao D. et al., 2018).

## Conclusion

The results obtained worldwide to date from the use of highly effective biotechnological methods deepen the understanding of the molecular mechanisms regulating the quality of rice grain. They provide theoretical support for molecular breeding to improve rice quality, in particular through the development of markers of economically valuable traits and germplasm resources for the genetic improvement of crop plants, including rice, for traits associated with the most important physical properties – vitreousness/chalkiness, shape and size of rice grain.

Hundreds of QTLs for the undesirable grain quality trait – chalkiness – have been identified in rice production centers worldwide. Only a few have been accurately mapped or sequenced, including Chalk5, a positively regulated factor expressed in the endosperm located on chromosome 5 and the first major QTL sequenced for chalky grain.

Additionally, the qPGWC-7, qPGWC-8, and qACE-9 QTLs were accurately mapped. Eight marker–trait associations were identified by screening genotypes with primers specific for rice grain size traits. Specific markers for length, width, thickness, grain length-to-width ratio, GS3RGS1, and RM505, were identified and can be used as markers of choice in breeding. Using DAP-seq of multiple rice accessions, it was shown that overexpression of OsFIF3 inhibits the expression of FLO2 and SUT1, thereby increasing grain chalkiness and reducing grain size. Three QTLs for grain shape (qGW3, qGL7.2, and qLWR7.1) and four QTLs for grain chalkiness (qWBR1.2, qBR2, qWCR3, and qWCR11) were identified (Liu J. et al., 2024). Numerous genes associated with grain quality were shown to influence both grain size and chalkiness in rice.

Thus, the level of understanding of the rice genome worldwide is high, and increased attention is being paid to the most important physical parameters of the rice grain at global rice research centers. Research is an ongoing process with a clearly defined goal of achieving the highest possible level of the best-quality rice products.

## Conflict of interest

The authors declare no conflict of interest.
